# University Students’ Sleep and Mental Health Correlates in South Korea

**DOI:** 10.3390/healthcare10091635

**Published:** 2022-08-27

**Authors:** Jinyoung Kim, Eun Hee Hwang, Sujin Shin, Kon Hee Kim

**Affiliations:** 1 School of Nursing, University of Nevada, Las Vegas, NV 89154, USA; 2 Department of Nursing, Wonkwang University, Iksan 54538, Korea; 3 College of Nursing, Ewha Womans University, Seoul 03760, Korea

**Keywords:** university student, sleep, depression, adult ADHD

## Abstract

Sleep closely relates to emotional instability. Recent studies report an increase in young adults’ poor sleep and associated mental health problems, including attention deficit hyperactivity disorder (ADHD), anxiety, depression, and so on. However, the information on related modifiable factors of these variables is still lacking. This cross-sectional study examined the association of sleep patterns and sleep quality with ADHD and depression in university students. A total of 290 participants aged 18–27 (Mean = 22.0, SD = 2.1) completed a structured questionnaire consisting of the Pittsburgh Sleep Quality Index (PSQI), the Adult ADHD, and the Center for Epidemiologic Studies Depression scales (CES-D). Of the participants, more than half (52.7%) slept 6 to 8 h per night, and 37% slept less than 6 h. Only 10% reported they went to bed before midnight; 40% went to sleep after 2 am. The mean PSQI score was 5.9 (SD = 2.9) for total participants and significantly correlated with ADHD scores and with depression scores. After adjusting for covariates, PSQI significantly aligned with increased risk for ADHD (β = 0.29, *p* = 0.036) and depression (β = 0.67; *p* < 0.001). Late bedtime was a significant factor for depression only. Sleep quality rather than sleep pattern significantly related to ADHD scores, whereas both sleep quality and bedtime aligned with depression scores. Additional studies are needed to develop strategic interventions for university students with ADHD and depression as well as underlying mechanisms.

## 1. Introduction

Sleep health at night is fundamental behavior related to daytime functioning, emotional status, and the pathophysiology of various diseases. Over the past decades, sleep durations are getting shorter, and bedtimes are getting later for university students. Thus, the prevalence of disrupted sleep is gradually increasing [1]. However, which factor—sleep quantity or quality—has a more significant impact on prevalent behavioral problems such as attention-deficit hyperactivity disorder (ADHD) and depression remains unclear.

Young adults, especially university students, have reported poor sleep and its negative impact on their life [2]. Because they should adapt to campus life, which includes both an individual’s internal adjustment and adaptation to the external environment, along with entering the university, In the process, many of them are dissatisfied with their campus life and suffer from problems related to career, academics, economic status, and interpersonal and heterosexual relationships [3,4,5]. This can negatively affect various aspects, such as the physical and psychological health of university students. The authors decided to include the major as a general characteristic based on a research finding indicating that depression and sleep levels were poorer in healthcare-related majors than in other majors [6]. The association between ADHD and depression has been continuously reported, and significant differences according to general characteristics such as gender and age have been confirmed [7]. The sleep of ADHD patients also causes the worsening of symptoms as well as difficulties in maintaining daily life, and it is reported that they are affected by various socioeconomic factors [8,9,10]. The rate of ADHD symptoms experienced in young adults is increasing. In the case of adolescents with ADHD, the more severe the depression, the higher the suicide intention [11]. Adult ADHD patients can experience trauma and safety jeopardy due to sleep disturbance [12,13]. However, fewer studies examined young adults’ sleep and mental health correlates, including ADHD, depression, and so on. Most of them considered young adults’ sleep as the quality of sleep only. Considering previous research results, examining the relationship between sleep, including quality and patterns, and its impact on mental health, including ADHD and depression symptoms is necessary.

The symptoms of ADHD, which are considered frequent behavioral issues in children, include inattention, impulsivity, and hyperactivity [14]. However, recent longitudinal studies reported that ADHD symptoms in childhood lasted until adulthood in 30–50% of ADHD patients [15,16]. The prevalence of ADHD in adults is known to be 2–8% [17], similar to that in children, which is 4–5% [18]. ADHD symptoms manifesting in adulthood result in difficulties in daily life, school/work performance, accidents, and interpersonal relationships [19,20,21]. Moreover, ADHD symptoms often lead to other behavioral and psychological problems such as depression, suicide attempts, and alcohol addiction [22,23]. Although the prevalence of ADHD tends to increase over decades and growing evidence indicates an adverse impact of ADHD in the young-adult population, information on factors aggravating ADHD behaviors and strategies for symptom management remains limited.

Until the Diagnostic and Statistical Manual of Mental Disorders-III (DSM-III), sleep problems were included in the diagnostic category of ADHD [24], and some studies have reported associations between sleep disorders and ADHD. It has been reported that the quantity and quality of sleep decrease in the presence of ADHD, and that it disturbs the circadian rhythm, which significantly affects sleep [25]. In particular, sleep shows significant interaction with the degree of ADHD symptoms, and sleep disturbance in ADHD patients can lead to the worsening of ADHD symptoms [8,9]. The interaction of adult ADHD with impulsivity and sleep may cause safety problems [12], but studies on sleep problems and related interventions in adult ADHD patients are very limited.

Depression is also a common psychological problem affecting approximately 30% of university students, which is higher than the general adult population [26]. In a couple of studies, among 30.7% of university students who reported they felt depressed during the past year, less than 50% sought care [27,28]. Previous studies showed a bidirectional relationship between sleep disturbances and depression [29,30,31]. Poor sleep quality or insomnia symptoms such as difficulty initiating or maintaining sleep are common in patients with depression [32,33]. Some researchers insist that sleep disturbances have a causal effect on depression [34]. However, research on the possible impact of sleep and sleep interventions on depression in university students is relatively few. For university students who are highly likely to be negatively affected by sleep due to stress related to academic achievement, career preparation, and intimate relationships [4], it is necessary to check the association between sleep and depressive symptoms. In particular, it should be considered that the results of previous studies show that ADHD symptoms negatively affect sleep and emotion in children with ADHD, and that these negative effects continue to aggravate ADHD symptoms [35]. Due to the characteristics of adults with ADHD that the symptoms are not as distinct as those of children with ADHD [36], studies on adults with ADHD are rare, so it is very significant to examine the association between ADHD symptoms, sleep, and depressive symptoms. Thus, the present study aimed to examine the association between sleep patterns and quality with ADHD and depression symptoms in university students.

## 2. Materials and Methods

### 2.1. Design

This descriptive and correlation study aimed to identify the association between sleep patterns and quality with symptoms of ADHD and depression in university students.

### 2.2. Participants

The study sample comprised 290 students from three universities located in three cities by purposive quota sampling. To maintain middle effect size f^2^ = 0.15, power 0.95, *p*-value 0.05, and 20 predictive variables, 222 participants were calculated using the G*Power program 3.1.9.2 (Allgemeine Psychologie und Arbeitspsychologie of Heinrich Heine Universtät, Düsseldorf, Germany) for the multiple linear regression [37]. A total of 300 students were selected, considering the dropout rate of 20%. One hundred questionnaires were distributed to each of the three selected universities, and 291 students completed the questionnaire (response rate 97.0%); then, one student with insufficient responses was excluded during the data analysis process.

### 2.3. Measurements

#### 2.3.1. Sleep Patterns and Quality

To assess sleep patterns, the authors asked about the average amount of sleep (<6, 6–7, 7–8, or 8–9 h per night), time to bed (before midnight, 0–1 am, 1–2, and after 3 am), and time of awakening (<6 am, 6–7, 7–8, and after 9 am). We used the Pittsburgh Sleep Quality Index (PSQI) to measure overall sleep quality [38]. The PSQI is a self-rated questionnaire that assesses subjective sleep quality, including sleep latency, habitual sleep efficiency, sleep disturbances, use of sleeping medication, and daytime function over the previous 1 month. The sum of scores is 19 items, each weighted equally on a 0–3 scale, yielding a global score ranging from 0 to 21. Higher scores indicate worse sleep quality.

#### 2.3.2. Adult ADHD 

We used the Adult Attention Deficit and Hyperactivity Disorder Scale (AADHDS) by Murphy and Barkley to measure participants’ ADHD symptoms [39]. This scale has been translated into Korean and validated [40]. This self-rating scale consists of nine questions that measure lack of attention and nine questions about hyperactivity and impulsiveness, for a total of 18 questions. The scale evaluates the presence of ADHD symptoms and assesses how often the symptoms have occurred over the preceding six months, with a 4-point scale ranging from “almost never” to “very often.” In the study [40] validating the tool, Cronbach’s α was 0.85, and it was 0.76 in the present study. 

#### 2.3.3. Depression

Depression was measured using the Center for Epidemiologic Studies Depression (CES-D) developed by Radolff [41]. The CES-D has been translated into Korean and validated [42]. It is a 20-item measure that asks participants to rate how often they experienced symptoms associated with depression over the past week, such as restless sleep, poor appetite, and feeling lonely. Each question could be answered by “rarely or none of the time” for 0 points, “some or little of the time” for 1 point, “moderately or much of the time” for 2 points, or “most or almost all the time” for 3 points. Thus, the total score ranged from 0–60 points, with high scores indicating greater depressive symptoms. Further, a score of 16 points or higher is indicative of clinical depression. In the study developing the tool, Cronbach’s α ranged from 0.85 to 0.90. In this study, Cronbach’s α was 0.89.

#### 2.3.4. Other Variables

We examined demographic and socioeconomic factors (i.e., age, gender, academic grade, major, residential type, part-time job, etc.). In terms of school performance, we asked about satisfaction with general campus life and major as well as participants’ academic achievement using their grade point average (GPA). In addition, items about drinking and smoking habits, exercise, and personality were asked. Participants answered the personality item with options, extrovert versus introvert, by their perception of their personality.

### 2.4. Data Collection

After approval by the IRB, data were collected from three universities located in three urban areas in South Korea. The authors received approval and cooperation from several departments of each university in advance and posted a notice of participant recruitment on the announcement board of each department. Three hundred students who read the notice and decided to participate, gathered at specific places prepared in each university. After explaining the purpose and procedure of our study, 291 students understood the purpose, process, and contents of the study and provided written informed consent to participate spontaneously in the study. All students who submitted consent forms completed a self-reported structured questionnaire consisting of AADHDS, CES-D, PSQI, demographic, and socioeconomic characteristics. It took about 20 min to respond, and after submitting the questionnaire, a small gift was provided. During the data analysis process, we excluded one questionnaire because of insufficient response.

### 2.5. Data Analysis

Collected data were analyzed using the SAS 9.4 program (SAS Institute, Cary, NC, USA). The authors summarized general characteristics using descriptive statistics with frequency and percentage. The difference in the degree of ADHD and depression according to the participants’ general characteristics was analyzed using a t-test and one-way ANOVA, followed by Scheffé’s post-hoc tests. We analyzed PSQI scores with AADHDS and CES-D scores using Pearson’s correlation coefficients to identify correlations among ADHD, sleep quality, and depression. We used a stepwise multiple linear regression analysis to examine sleep characteristics and the factors influencing adult ADHD and depression symptoms.

## 3. Results

### 3.1. General Characteristics

Table 1 summarizes the general characteristics of the participants. The mean age of participants was 22.0 (±2.1) years; 68% of the 290 were women. Approximately 57% of participants majored in health science, including nursing. Most participants lived with their parents or in a dormitory and had GPAs equal to or greater than 3.0. Those who reported they drank alcohol and smoked cigarettes were 65.5% and 8.7%, respectively. One of four did a regular exercise in this sample. Only 4% of participants reported they were dissatisfied with their major; 7% were dissatisfied with campus life.

### 3.2. Participants’ Characteristics Associated with ADHD and Depression

We compared ADHD and depression scores among subgroups of general characteristics in Table 1. The ADHD scores differed significantly by major (*F* = 3.25; *p* = 0.013) and GPA (*F* = 3.15; *p* = 0.025). ADHD scores for those majoring in health science except for nursing were significantly higher (*p* < 0.05) than those in nursing and engineering.

Those who did not know/report their GPA had higher ADHD scores than students with GPA ≥ 3.0. Men studying education and those doing regular exercise had significantly lower depression scores than other groups. Depression scores were significantly higher in those dissatisfied with campus life compared with “satisfied” and “neutral” groups.

### 3.3. Sleep Patterns and Quality: Association with ADHD and Depression

Table 2 shows sleep patterns, such as sleep duration, bedtime, and awakening time. We then compared ADHD and depression scores among subgroups. Although almost half of the participants (52.7%) slept 6 to 8 h per night, 37% slept less than 6 h and 10% more than 8 h. In this sample, only 10% reported they went to bed before midnight; 40% initiated sleep after 2 am. The most frequent awakening time was between 7 and 8 am, and 20% got up after 9 am. We next compared ADHD and depression scores among subgroups. As shown in Table 2, depression scores differed significantly (*p* = 0.005) among bedtime subgroups only. Those who went to bed after 3 am showed significantly (*p* < 0.05) higher depression scores (18.5 ± 6.5) than those who slept before midnight (13.7 ± 5.5) and those in the midnight to 1 am group (13.6 ± 5.1).

The mean score for sleep quality, measured by the PSQI, was 5.9 (SD = 2.9, range 0–17) in total participants. We examined correlations between sleep quality, ADHD, and depression scores. The correlation between sleep quality and ADHD (r = 0.22; *p* < 0.001) and depression (r = 0.42; *p* < 0.001) was significant with weak to medium strength. ADHD and depression correlated significantly with weak strength (r = 0.24; *p* < 0.001).

### 3.4. Results by Multivariate Models

We performed a multivariate regression analysis to examine the independent associations of sleep patterns and quality with ADHD and depression scores. As shown in Table 3, after controlling for other significant covariates (major, academic grade, GPA, drinking habit, and satisfaction with campus life) after a univariate regression analysis, the PSQI significantly aligned with increased risk for ADHD scores (β = 0.293; *p* = 0.036) and depression scores (β = 0.673; *p* < 0.001). Bedtime was a significant factor for depression symptoms only. Night owls, those going to sleep after 3 am, independently aligned with depression symptoms (β = 3.594; *p* = 0.022). 

## 4. Discussion

The present study found short sleep duration and delayed bedtime in university students. Thirty-seven percent of the 290 students reported they slept less than 6 h, and only 10% went to bed before midnight. Their sleep quality was also poor, with an average score of 5.9, which is higher than the cut-off point for detecting poor sleep quality by the PSQI. After adjusting for other socioeconomic and lifestyle factors, sleep quality rather than sleep patterns was significantly related to ADHD scores, whereas bedtime and sleep quality both aligned with depression scores.

In previous studies, researchers found sleep problems to be common in adult ADHD. Fisher and colleagues reported that 59.1% of 877 adults diagnosed with ADHD had 1 to 3 sleep problems, including trouble getting to sleep, unrefreshed or restless sleep, and frequent awakenings [43]. Sleep problems were age-dependent in their study: More prevalent in adult ADHD than in children. In addition to poor sleep quality, delayed sleep phases or irregular sleep patterns are often reported in individuals with ADHD [44,45,46]. However, the underlying mechanisms of sleep problems in ADHD remain unclear. Unfortunately, our data cannot address the potential mechanisms. A plausible explanation from previous studies is that prolonged sympathetic activation due to hyperactivity in ADHD can alter one’s internal biological clock, which regulates the sleep–wake cycle [47,48]. The internal biological clock in the hypothalamus is regulated mainly by light–dark exposure [49]. After sunset, therefore, the biological clock sends signals to different regions of brain that control hormonal and neural changes to initiate and maintain sleep throughout the night. However, other external stimuli such as food or caffeine/alcohol intake and prolonged activities at night can disturb normal sleep–wake cycles. Coogan and McGowan reported meta-analysis findings that interrupted circadian rhythms, including delayed sleep onset, eveningness, and so forth, associated with ADHD should be considered for ADHD treatments [50]. In previous studies, insomnia symptoms and evening preference were related to hyperactivity and other ADHD symptoms in young adults [51,52].

In the present study, however, only overall sleep quality, including frequent awakening, sleep efficiency, and use of sleep medicine, was significantly worse in students with ADHD symptoms than those without ADHD symptoms, whereas sleeping time and patterns such as bedtime and wake time were not significantly different. These research results support the results of previous studies that high school students with ADHD suffer from sleep disorders such as insomnia and daytime sleepiness due to ADHD symptoms [53]. On the other hand, it is different from the study results that children with ADHD experience changes in their sleep patterns due to ADHD symptoms, and these sleep changes exacerbate ADHD symptoms [35]. In other words, although ADHD patients are affected by sleep patterns, sleep quality, insomnia, daytime sleepiness, etc., sleep disorder types according to age, such as sleep patterns, are somewhat different. University students diagnosed with ADHD are experiencing maladaptation to college life and general functional impairment due to daytime sleepiness, which threatens their academic achievement [54]. Additionally, in this study, the participants had problems with frequent awakening or sleep efficiency, so it is necessary to find a way to maximize sleep quality by minimizing awakening during sleep.

Bidirectional relationships between depression and sleep disturbances have been relatively well known [33], although few studies were performed on university students. In some previous studies for university students, poor sleep hygiene and sleep quality, as well as insufficient social support and a history of mental health, aligned with increased risk for depression [55], whereas depression significantly predicted insomnia in other studies [56]. A recent prospective study also supported the predictive value of depression for sleep difficulty, showing that having depressive symptoms in adolescence was a moderate predictor for sleep disturbance in early adulthood. In contrast, the opposite relationship was not significant [57]. Regarding sleep patterns and sleeping time, Regestein and colleagues reported that weekday sleep debt, an indicator of insufficient sleep, might cause depression in college women [58], consistent with another study in South Korea in which researchers reported that sleep debt was an independent risk factor for suicide attempt [59]. A meta-analysis study [60], in which sleep disturbances, including insomnia and nightmares, are statistically significant risk factors for suicidal thoughts and behaviors, supported our results. In the present study, late bedtime, which may cause insufficient sleep, is aligned with depression. Similar to our findings, night owls who go to bed relatively late were more likely to be depressed compared to chronotypes in previous studies [51]. Moreover, stress-induced serotonin system changes may disrupt circadian rhythms and increase the vulnerability to depression [61]. Further studies are needed to confirm whether late bedtime alone can cause depression in university students or young adults. 

The present study had several limitations. First, as this study had a cross-sectional design, we could not determine the causal effect of sleep on ADHD and depression. Second, we measured sleep patterns and sleep quality using self-reported questionnaires. Even though the PSQI is the most widely used scale for sleep quality, some recall or response bias may influence the measurements. The present study also self-reported sleep duration, bedtime, and wake time. Objective measurement by sleep studies or actigraphy may provide more accurate information on sleep quantity in future studies. Third, we did not interview participants’ cohabitants who can observe their signs and symptoms of ADHD or depression, so our findings could be limited to symptoms. Studies using other instruments which measure ADHD or depression by significant others are recommended. Self-reported sleep and mental health problems could not mean clinical diagnoses. Thus, the present results should be carefully interpreted and applied. Fourth, the environmental factors on sleep, ADHD or depression, for example, social media addiction, were not considered. These factors should be examined in the following studies. Last, we used convenience sampling, which can undermine the representability and generalizability of study participants. We guess that students who were interested in their sleep and mental health and were expected to maintain a healthy lifestyle and relatively good mental health participated in our study. The students that did not participate might have more severe problems and withdraw socially. Further studies in different samples, including high-risk students and countries, are needed. 

## 5. Conclusions

University students are in the stage of transition from adolescence to adulthood in the developmental process. They are required to adapt to the university’s environment and the individual’s internal adaptation. They also experience various stresses while deciding on job and career paths and trying to achieve them. Stress causes or aggravates emotional problems such as low self-esteem and depression and negatively affects sleep, which is essential for a quality life. Students with ADHD symptoms are particularly affected. While studies that consider sleep or emotional aspects of university students with ADHD symptoms are rare, the present study showed that, for university students, how well they sleep at night rather than how much or when they sleep might be more important to managing ADHD behaviors. These findings are significant because they prepared relevant empirical data by identifying the association between university students’ sleep and mental health, including ADHD and depression. It is significant to develop and apply sleep-considering interventions for students with ADHD or depression, even both, based on our findings. Further research that supplements our limitations should be essential. Night owls tended to be more depressed in this study. Further studies are needed to confirm a causal relationship. At the same time, considering the increasing prevalence of ADHD, it is necessary to consider the development and application of psychosocial nursing interventions considering their sleep patterns along with repeated studies on the current status of university students with ADHD symptoms and their influencing factors. This will help university students smoothly transition into adulthood, and it is thought that attention will be paid to not only adjusting to college life but also planning a healthy lifestyle in the future.

## Figures and Tables

**Table 1:** Attention-Deficit Hyperactivity Disorder and depression scores by subgroups of general characteristics. (*n* = 290).

Characteristics		*n* (%)	ADHD ^1^ Mean ± SD ^2^	t or F (*p*)	Depression Mean ± SD ^2^	t or F (*p*)
Gender	Male	92 (31.7)	9.2 ± 7.3	1.25 (0.477)	14.1 ± 5.4	**2.38 (0.018)**
	Female	198 (68.3)	9.8 ± 6.2		15.8 ± 6.1	
Major	Engineering	59 (20.4)	9.1 ± 6.1	3.25 (0.013)	14.2 ± 5.7	**2.62 (0.036)**
	Administration	39 (13.5)	10.6 ± 6.4		16.6 ± 7.1	
	Education	26 (9.0)	9.5 ± 6.9		12.7 ± 5.3	
	Nursing	127 (43.9)	8.7 ± 5.9		15.7 ± 5.3	
	Other health science	38 (13.2)	12.8 ± 8.4		16.1 ± 5.8	
Academic grade	Freshman	73 (25.2)	9.2 ± 6.4	2.62 (0.051)	15.8 ± 5.9	1.50 (0.215)
	Sophomore	97 (33.5)	11.1 ± 7.2		15.4 ± 6.3	
	Junior	67 (23.1)	8.7 ± 6.3		15.7 ± 5.5	
	Senior	53 (18.3)	8.6 ± 5.5		13.8 ± 5.6	
Religion	Yes	124 (42.8)	9.5 ± 6.4	0.20 (0.840)	15.0 ± 6.1	0.87 (0.388)
	No	166 (57.2)	9.7 ± 6.9		15.6 ± 5.6	
Relationship	Yes	110 (38.2)	9.9 ± 6.6	0.60 (0.549)	15.0 ± 6.1	0.75 (0.452)
	No	178 (61.8)	9.4 ± 6.6		15.6 ± 5.6	
Living	With parents	126 (43.5)	10.3 ± 6.9	1.14 (0.334)	14.8 ± 5.6	0.52 (0.672)
	In a dormitory	88 (30.3)	9.3 ± 6.6		15.8 ± 5.7	
	Alone	73 (25.2)	9.1 ± 6.0		15.3 ± 6.6	
	Etc.	3 (1.0)	5.0 ± 1.0		15.7 ± 7.2	
Grade point average (GPA)	≥4.0	27 (9.3)	8.1 ± 5.5	3.15 (0.025)	16.0 ± 5.1	0.85 (0.468)
	3.0–3.9	200 (69.0)	9.2 ± 5.9		15.0 ± 5.6	
	less than 3.0	60 (20.7)	11.4 ± 8.5		15.6 ± 7.3	
	don’t know	3 (1.0)	15.3 ± 8.5		19.3 ± 3.1	
Part-time job	Yes	68 (23.7)	9.4 ± 4.8	0.30 (0.764)	14.9 ± 5.0	0.67 (0.506)
	No	219 (76.3)	9.6 ± 7.1		15.4 ± 6.2	
Drinking	Yes	190 (65.5)	9.7 ± 6.7	0.34 (0.731)	15.6 ± 6.1	1.37 (0.171)
	No	100 (34.5)	9.4 ± 6.4		14.6 ± 5.5	
Smoking	Yes	25 (8.7)	9.3 ± 6.1	0.24 (0.807)	14.1 ± 6.6	1.04 (0.301)
	No	263 (91.3)	9.6 ± 6.6		15.4 ± 6.8	
Exercise	Yes	78 (27.2)	9.2 ± 6.6	0.51 (0.610)	14.0 ± 4.8	2.51 (0.013)
	No	209 (72.8)	9.7 ± 6.3		15.7 ± 6.2	
Satisfaction with major	Satisfied	165 (56.9)	9.4 ± 6.9	0.17 (0.841)	14.9 ± 5.7	0.64 (0.528)
	Neutral	113 (39.0)	9.9 ± 6.3		15.6 ± 6.2	
	Dissatisfied	12 (4.1)	9.3 ± 4.2		16.3 ± 6.7	
Campus life	Satisfied	144 (49.7)	8.7 ± 6.2	2.84 (0.060)	14.4 ± 5.8	6.36 (0.002)
	Neutral	125 (43.1)	10.6 ± 6.8		15.6 ± 5.4	
	Dissatisfied	21 (7.2)	9.7 ± 7.4		19.1 ± 8.0	
Self-determined personality	Introverted	141 (49.0)	9.1 ± 6.5	1.25 (0.213)	14.1 ± 5.4	0.66 (0.507)
	Outgoing	147 (51.0)	10.1 ± 6.7		15.8 ± 6.1	

^1^ Attention-Deficit Hyperactivity Disorder. ^2^ Standard Deviation.

**Table 2:** Comparison of ADHD and depression scores among subgroups of sleep patterns. (*n* = 290).

Variable		*n* (%)	ADHD ^1^		Depression	
			Mean ± SD ^2^	*p*	Mean ± SD ^2^	*p*
Sleep duration	<5 h	44 (15.2)	10.0 ± 6.5	0.817	16.4 ± 6.0	0.185
	5–6	64 (22.1)	9.7 ± 7.5		16.0 ± 5.9	
	6–7	79 (27.2)	10.0 ± 6.0		15.4 ± 5.9	
	7–8	74 (25.5)	9.3 ± 6.1		14.1 ± 5.4	
	8≤	29 (10.0)	8.5 ± 7.4		14.6 ± 6.9	
Bedtime	<midnight	30 (10.4)	9.2 ± 7.4	0.938	13.7 ± 5.5	0.005
	0–1 am	56 (19.4)	9.1 ± 6.3		13.6 ± 5.1	
	1–2	87 (30.1)	10.0 ± 6.9		15.3 ± 6.0	
	2–3	93 (32.2)	9.6 ± 6.5		16.1 ± 5.9	
	3≤	23 (8.0)	9.7 ± 5.9		18.5 ± 6.5	
Awakening time	<7 am	57 (19.7)	9.8 ± 7.4	0.568	15.5 ± 5.9	0.347
	7–8	102 (35.2)	10.1 ± 6.5		15.1 ± 5.5	
	8–9	73 (25.2)	8.7 ± 6.0		14.5 ± 5.8	
	9≤	58 (20.0)	9.8 ± 6.4		16.3 ± 6.7	

^1^ Attention-Deficit Hyperactivity Disorder. ^2^ Standard Deviation.

**Table 3:** Association of sleep quality and bedtime with ADHD and depression by multivariate regression analysis. (*n* = 290).

Variable		ADHD ^1^				Depression			
		β	SE ^3^	t	*p*	β	SE ^3^	t	*p*
PSQI ^2^ total score		0.293	0.14	2.11	0.036	0.673	0.11	6.07	<0.001
Bedtime	<midnight	Reference				Reference			
	0–1 am	0.308	1.44	0.21	0.831	−0.824	1.26	−0.65	0.515
	1–2	0.597	1.36	0.44	0.660	0.852	1.19	0.72	0.474
	2–3	0.326	1.34	0.24	0.808	0.852	1.18	1.10	0.272
	3≤	−0.004	1.79	−0.00	0.998	3.594	1.56	2.30	0.022

^1^ Attention-Deficit Hyperactivity Disorder. ^2^ Pittsburgh Sleep Quality Index. ^3^ Standard Error.

## Data Availability

Not applicable.
